# Mechanosensitive Ion Channel Piezo1 Regulates Diet-Induced Adipose Inflammation and Systemic Insulin Resistance

**DOI:** 10.3389/fendo.2019.00373

**Published:** 2019-06-13

**Authors:** Can Zhao, Qiushi Sun, Lingyi Tang, Yang Cao, Jamison L. Nourse, Medha M. Pathak, Xiang Lu, Qin Yang

**Affiliations:** ^1^Department of Geriatrics, The Affiliated Sir Run Run Hospital of Nanjing Medical University, Nanjing, China; ^2^Department of Medicine, Physiology and Biophysics, UC Irvine Diabetes Center, University of California at Irvine, Irvine, CA, United States; ^3^Department of Geriatrics, The Second Affiliated Hospital, Nanjing Medical University, Nanjing, China; ^4^Key Laboratory for Aging and Disease, Nanjing Medical University, Nanjing, China; ^5^Department of Cardiology, The Affiliated Hospital of Nanjing University of Chinese Medicine, Nanjing, China; ^6^Department of Physiology and Biophysics, Sue and Bill Gross Stem Cell Research Center, Center for Complex Systems Biology, University of California at Irvine, Irvine, CA, United States

**Keywords:** adipocytes, insulin sensitivity, Piezo1, inflammation, lipolysis, TLR4

## Abstract

Adipocytes function as an energy buffer and undergo significant size and volume changes in response to nutritional cues. This adipocyte plasticity is important for systemic lipid metabolism and insulin sensitivity. Accompanying the adipocyte size and volume changes, the mechanical pressure against cell membrane also changes. However, the role that mechanical pressure plays in lipid metabolism and insulin sensitivity remains to be elucidated. Here we show that Piezo1, a mechanically-activated cation channel stimulated by membrane tension and stretch, was highly expressed in adipocytes. Adipose Piezo1 expression was increased in obese mice. Adipose-specific piezo1 knockout mice (adipose-Piezo1^−/−^) developed insulin resistance, especially when challenged with a high-fat diet (HFD). Perigonadal white adipose tissue (pgWAT) weight was reduced while pro-inflammatory and lipolysis genes were increased in the pgWAT of HFD-fed adipose-Piezo1^−/−^ mice. The adipose-Piezo1^−/−^ mice also developed hepatic steatosis with elevated expression of fatty acid synthesis genes. In cultured adipocytes, Piezo1 activation decreased, while Piezo1 inhibition elevated pro-inflammatory gene expression. TLR4 antagonist TAK-242 abolished adipocyte inflammation induced by Piezo1 inhibition. Thus, adipose Piezo1 may serve as an adaptive mechanism for adipocyte plasticity restraining pro-inflammatory response in obesity.

## Introduction

Adipocytes are responsible for storing food energy in the form of fat. This function demands the cells to be highly plastic and dynamic in response to nutritional cues. When nutrients are sufficient, adipocytes increase their size and volume for fat storage. Under the condition of limited nutrient availability, adipocytes undergo lipolysis to release fat to provide energy for other organs (1, 2). The balance between fat storage and lipolysis plays a major role in regulating systemic lipid metabolism and insulin sensitivity. In obesity, adipose plasticity is impaired and the balance of fat storage and lipolysis is disrupted, leading to adipose inflammation and enhanced lipolysis, which in turn cause systemic insulin resistance due to ectopic lipid deposition in other metabolic organs such as liver (3, 4). Insulin resistance is an independent risk factor for type 2 diabetes, cardiovascular disease and multiple cancers (5–8).

The molecular mechanisms for the impaired adipocyte plasticity in obesity are not well understood. The increased triglycerides in adipocytes in obesity apply significant pressure to the plasma membrane from inside out. At the meantime, adipose fibrosis developed in obesity also exerts pressure from outside in. Therefore, the plasma membrane of adipocytes is under constant mechanical pressures, which make adipocytes “stiffer” and less plastic. Adipocyte stiffness is one major mechanism for adipose inflammation and systemic insulin resistance in obesity (9–11). However, the mechanistic link between adipocyte mechanical pressure and plasticity-mediated inflammation and insulin resistance remains to be elucidated.

Piezo1 is recently identified as a novel mechanically-gated ion channel that transduces mechanical stimuli into electrical and chemical signals in mammalian cells (12, 13). Piezo1 is highly expressed in tissues with high mechanical pressures and plays an essential role in vascular development (14, 15), blood pressure regulation (16), and red blood cell volume control (17, 18). Piezo1 expression is also increased in bladder carcinoma (19), glioma (20), and breast cancer (21). However, the roles of Piezo1 in regulating adipocyte function have not been reported. In the current study, we found that Piezo1 is highly expressed in adipose tissue. Deletion of Piezo1 specifically in adipocytes reduces perigonadal fat mass and causes adipose inflammation, insulin resistance, and hepatic steatosis. Therefore, Piezo1 plays a key role in regulating adipose plasticity and insulin resistance in obesity.

## Materials and Methods

### Mice

Adiponectin-Cre mice were purchased from the Jackson Laboratory (Stock No: 010803). The Piezo1-flox and Piezo1-tdTomato were generously provided by Dr. Ardem Patapoutian at The Scripps Research Institute. To generate adipose-specific Piezo1 knockout mice, Piezo1-flox/flox mice were crossed to adiponectin-Cre mice. Mice were housed four or five per cage and maintained under a 12 h light/12 h dark cycle at constant temperature (23°C) with *ad libitum* access to normal laboratory chow (#2920X; Harlan Teklad) and water. For diet-induced obesity, mice were fed a high-fat diet containing 54.8% fat calories, 24.0% carbohydrate calories, and 21.2% protein calories (4.8 kcal/g) (TD.93075; Envigo Inc.) from 4 weeks of age for 30–35 weeks. Body weight was measured biweekly. All mouse studies were conducted in accordance with federal guidelines and were approved by the Institutional Animal Care and Use Committee of the University of California, Irvine.

### GTT and ITT

For the glucose tolerance test (GTT) and insulin tolerance test (ITT), food was removed at 10:00 a.m. GTTs were performed 5 h after food removal by the intraperitoneally (i.p.) injecting glucose at 1 g/kg of body weight. ITTs were performed with i.p. injections of recombinant regular human insulin (Novolin R, ReliOn) at a dose of 0.75 to 1.2 U/kg of body weight. Blood glucose was measured at the indicated time points (One Touch Ultra glucometer, Johnson and Johnson).

### Serum Insulin, NEFA and Triglyceride Measurements

Serum insulin was measured with Ultrasensitive Insulin ELISA kit (90080, Crystal Chem). Serum non-esterified fatty acids (NEFA) were measured using NEFA Assay kit (276-76491, Wako). Total lipid from liver was isolated using Folch method (22). Serum and tissue triglyceride were measured using L-Type triglyceride Assay kit (461-09092, Wako). For the stimulated lipolysis, NEFA was measured in serum taken from mice 20 min after intraperitoneal injections of β3-adrenergic agonist CL 316,243 (C5976, Sigma, 1 mg/kg).

### Mouse Primary SVF and Adipocytes Isolation and Cell Culture

Mouse subcutaneous inguinal fat pads were removed and washed with PBS pH7.4 (Gibco) and then minced. The minced tissue was digested for 15–20 min at 37°C in the digestion buffer (10 mM CaCl_2_, 2.5 unit/ml collagenase D, 2.4 units/ml Dispase II in PBS). The digested tissue was filtered through a 100 μm mesh and centrifuged at 600 × G for 5 min. Floating adipocyte fraction was removed. The resulting pellets were resuspended and further filtered through the 40 μm nylon cell strainers (BD Biosciences). Stromal vascular fraction (SVF) cells were maintained in DMEM/F12 GlutaMAX (Invitrogen) containing 15%FBS, 100 U/mL penicillin, and 100 μg/mL streptomycin. For differentiation, confluent preadipocytes were treated with medium containing 15% FBS, 0.5 mM isobutylmethylxanthine (I7018; Sigma), 1 μM dexamethasone (D4902; Sigma), 2 μg/mL insulin (I0546; Sigma), and 1 μM rosiglitazone (R2408; Sigma) for 48 h. Adipocytes were then maintained in medium containing 15% FBS and 2 μg/mL insulin. After 6–7 days of induction, differentiated cells were challenged with 5 or 20 μM Yoda1 (SML1558, Sigma), 1 μM TAK-242 (5.08336, Millipore) and 5 μM GsMTx-4 (STG-100, Alomone) or vehicle for 18–20 h. Cells were collected for further analysis.

### Tissue Harvest and Histology

Mice were fasted for 5 h before sacrifice. Tissues were carefully dissected to avoid contamination from surrounding tissue. Samples for RNA and protein analysis were frozen immediately in liquid nitrogen and stored at −80°C for further studies. For histology, fat and liver samples were fixed in 10% buffered formalin and embedded in the paraffin wax. A Leica AutoStainer XL (Leica Biosystems Inc., Buffalo Grove, IL) was used for automated hematoxylin and eosin staining (H&E). The histology of adipose and liver tissue (*n* = 3–5 per group) was examined in 4–6 μm thick H&E stained sections. Adipocyte size was measured using NIH ImageJ software (23). A total of 243–478 adipocytes per mouse were measured. Briefly, the adipose tissue imaging was calibrated for background substation. The excess noise was removed and the threshold was set for defining areas consisting of membrane material and empty space identified by black and white, respectively. To help define the membranes of the individual adipocytes, each membrane is uniformly enhanced. The adipocyte area was then measured using the “Measure and Label Macro” for imageJ.

### Western Blot

Tissues or cells were lysed in radioimmunoprecipitation assay (RIPA) buffer containing 1 mM NaF, 1 mM sodium orthovanadate, and 1 mM phenylmethylsulfonyl fluoride. An equal amount of total protein (25 μg/lane for cells, 50 μg/lane for tissues) was loaded into 3–8% NuPAGE Tris-acetate gels (Invitrogen) and transferred to poly-vinylidene fluoride membranes for detection with indicated antibodies. The following antibodies were used: rabbit anti-RFP (600-401-379, Rockland) at 1:1,000 dilution; mouse anti-GADPH (60004-1-Ig, Proteintech) at 1:5,000 dilution.

### RNA Extraction and Quantitative PCR

Total RNA was extracted using the NucleoSpin^®^ RNA kit (740955, Macherey-Nagel). cDNA was synthesized using the SuperScript III first-strand synthesis supermix (11752, Invitrogen) for quantitative RT-PCR and used in real-time PCRs with Power SYBR Green PCR master mix (Applied Biosystems) on a 7900HT real-time PCR system (Applied Biosystems). The relative gene expression levels were calculated by the 2Ct method using Tata-binding protein (Tbp) or 36b4 as an internal control. Primer sequences are shown in Supplemental Table 1.

### Statistical Analysis

Data are presented as mean ± SEM and were analyzed by unpaired two-tailed Student *t*-test, repeated-measures ANOVA or one-way ANOVA, as appropriate. Statistical significance was assumed at *p* < 0.05.

## Results

### Piezo1 Is Highly Expressed in Adipocytes

**Figure 1:**
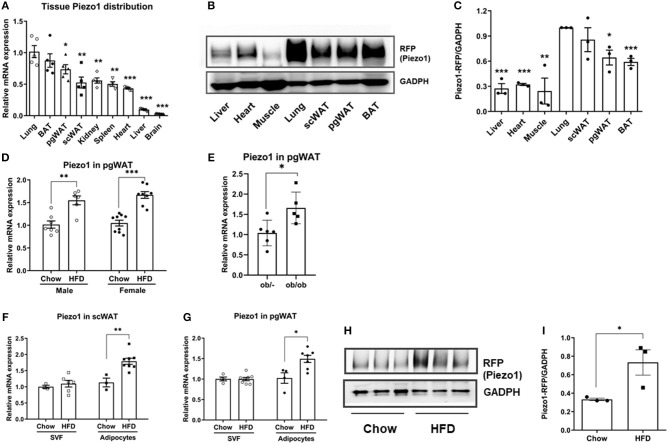
Piezo1 expression in tissues, isolated adipocytes and SVFs. **(A)** Tissue distribution of Piezo1 mRNA levels normalized to lung in chow-fed male mice (*n* = 5). **(B,C)** Piezo1-RFP protein expression in male mice fed a chow diet. Relative protein levels were normalized to GADPH. (*n* = 3, lung was set as 1). **(D)** Piezo1 mRNA expression in pgWAT of chow and HFD-fed mice (*n* = 6–9), all values were normalized to chow-diet fed mice. **(E)** Piezo1 mRNA in pgWAT of ob/ob and control mice (*n* = 5–6). **(F,G)** Piezo1 mRNA in adipocytes and SVF isolated from pgWAT and scWAT of chow or HFD-fed male mice (*n* = 3–8). All values were normalized to Chow-SVF. **(H,I)** Piezo1 protein expression in adipocytes isolated from pgWAT of chow and HFD-fed male mice. Relative protein levels were normalized to GADPH (*n* = 3). ^*^P< 0 .05, ^**^P < 0.01, ^***^P < 0.001. mRNA expression was corrected to Tbp. Data are expressed as mean ± SEM. SVF, stromal vascular fraction; pgWAT, perigonadal white adipose tissue; scWAT, subcutaneous white adipose tissue; BAT, brown adipose tissue; HFD, high fat diet; Tbp, TATA-Box Binding Protein.

To investigate the roles of Piezo1 in regulating adipose function, we first measured Piezo1 expression. Real-time qPCR showed that mouse Piezo1 mRNA levels were higher in brown adipose tissue (BAT), perigonadal white adipose tissue (pgWAT) and subcutaneous white adipose tissue (scWAT) than that in other organs except for lung (Figure 1A). Consistently, database searches revealed that Piezo1 mRNA in adipose tissue appeared to be the highest among 37 human tissues in the Human Protein Atlas RNA-seq data analysis (Supplementary Figure 1). Similarly, large-scale analyses of the mouse transcriptome (GSE9954) also showed that Piezo1 was highly expressed in adipose tissue (Supplementary Figure 2). Since there was no reliable Piezo1 antibody to detect Piezo1 protein, we measured the Piezo1-tdTomato fusion protein controlled by the endogenous Piezo1 promoter in the Piezo1-tdTomato transgenic mice using an RFP antibody (24). Consistent with the mRNA expression, Piezo1 protein was most abundant in lung, followed by scWAT, pgWAT, and BAT when normalized to GAPDH (Figures 1B,C). Western blot showed relatively diffuse bands which may be caused by Piezo1 posttranslational modifications (25–27).

We then investigated whether adipose Piezo1 would be regulated in obesity. Piezo1 mRNA levels in pgWAT were elevated in both male and female HFD-fed mice and in the morbidly obese ob/ob mice compared with controls (Figures 1D,E). We further separated adipocytes from SVF in adipose tissue and measured Piezo1 mRNA levels. Piezo1 expression was increased in adipocytes but not in SVFs isolated from pgWAT and scWAT of HFD-fed obese mice compared with that from chow-fed mice (Figures 1F,G). Consistently, when the Piezo1-tdTomato reporter mice were placed on a HFD, Piezo1-tdTomato protein in adipocytes was also elevated (Figures 1H,I). We also measured the expression of Piezo2, a homolog of Piezo1. Piezo2 mRNA was very abundant in lung, but the expression was much lower in other organs including adipose tissue (Supplementary Figure 3A). Although direct comparison was not possible, Piezo2 expression was likely much lower than Piezo1 in adipose tissue because the cycle threshold (Ct) values of Piezo2 were about 5 cycles higher than that of Piezo1 using three different sets of primers in the qPCR assays (not shown). Interestingly, adipose Piezo2 was also upregulated in obesity (Supplementary Figures 3B–D). These results show that Piezo1 is highly expressed in adipocytes and the expression is elevated in obesity.

### Adipose-Specific Piezo1 Knockout Mice Are Insulin Resistant

**Figure 2:**
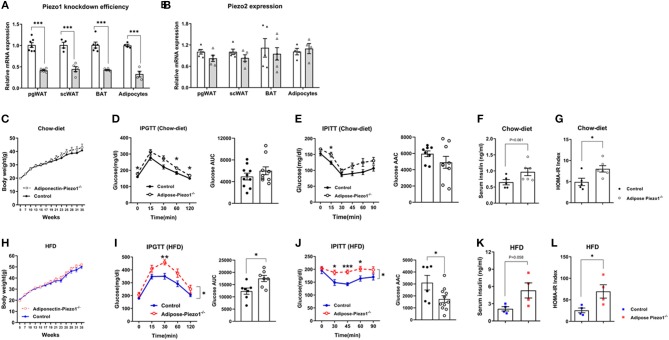
Adipocyte-specific Piezo1 knockout causes insulin resistance and glucose intolerance. **(A)** Piezo1 and **(B)** Piezo2 mRNA in pgWAT, scWAT, BAT, and adipocytes isolated from pgWAT (White bars: control mice; gray bars: adipose-Piezo1^−/−^ mice, mRNA expression was normalized to 36B4 as an internal reference gene, *n* = 4–7). **(C)** Body weight of adipose-Piezo1^−/−^ and control mice fed a chow diet (*n* = 5–6). **(D–G)** Intraperitoneal glucose tolerance test (IPGTT) with calculated area under the curve (AUC) **(D)**, insulin tolerance test (IPITT) with calculated area above the curve (AAC) **(E)** (*n* = 8–10), serum insulin levels **(F)** and HOMA-IR **(G)** (*n* = 5–6) in chow-fed adipose-Piezo1^−/−^ and control mice. **(H)** Body weight of adipose-Piezo1^−/−^ and control mice fed a high-fat-diet (*n* = 9–10). **(I–L)** IPGTT with calculated area under the curve (AUC) (*n* = 6–7) **(I)**, IPITT with calculated area above the curve (AAC) (*n* = 6–11) **(J)**, serum insulin levels **(K)** and HOMA-IR **(L)** (*n* = 4) in HFD-fed adipose-Piezo1^−/−^ and control mice. All data were collected from male mice. ^*^P < 0.05, ^**^P < 0.01, ^***^P < 0.001 vs. control group. Data are expressed as mean ± SEM. pgWAT, perigonadal white adipose tissue; scWAT, subcutaneous white adipose tissue; BAT, brown adipose tissue; HFD, high-fat diet; HOMA-IR, homeostasis model assessment of insulin resistance.

We generated adipose-specific Piezo1 knockout mice (adipose-Piezo1^−/−^) by crossing Piezo1-flox/flox mice to adiponectin-Cre mice (28, 29). Piezo1 expression was reduced by approximately 60% in adipose tissue and by about 70% in isolated adipocytes from adipose-Piezo1^−/−^ compared with that from control mice (Figure 2A). The expression of Piezo2 was not altered by Piezo1 knockout (Figure 2B). Body weight (Figures 2C,H), body weight gain (Supplementary Figure 4A), and body composition (Supplementary Figure 4B) were not different between adipose-Piezo1^−/−^ and control mice under either chow-fed or HFD-challenged conditions. Chow-fed adipose-Piezo1^−/−^ mice showed mildly elevated glucose levels in the intraperitoneal glucose tolerance test (GTT) and insulin tolerance test (ITT), but the areas under (GTT), or above (ITT) curves were not different (Figures 2D,E). The fold changes corrected to the basal glucose in GTT and ITT were comparable between chow-fed adipose-Piezo1^−/−^ mice and controls (Supplementary Figures 5A,B). Nevertheless, the chow-fed adipose-Piezo1^−/−^ mice may be mildly insulin resistant since insulin levels tended to be higher and HOMA-IR index was elevated (Figures 2F,G). When challenged with a HFD, adipose-Piezo1^−/−^ mice showed exacerbated insulin resistance as evidenced by significantly increased glucose levels in GTT and ITT, and elevated insulin and HOMA-IR index (Figures 2H–L). These data indicate that adipose Piezo1 is required for maintaining systemic glucose homeostasis and insulin sensitivity.

### Adipose Piezo1 Knockdown Reduces Perigonadal Fat Mass

**Figure 3:**
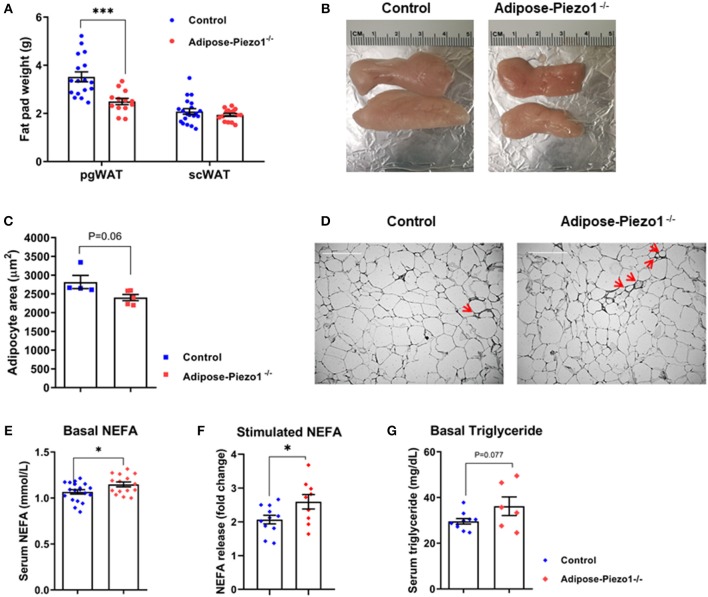
Effects of adipose Piezo1 knockout on adipocyte size and inflammation. **(A)** Fat pad weight of pgWAT and scWAT of HFD-fed adipose-Piezo1^−/−^ and control mice (*n* = 16–20). **(B)** Representative pgWAT pads. **(C)** Adipocyte area of pgWAT (*n* = 5). **(D)** H&E staining of pgWAT. Arrow: crown-like structures. Scale Bar, 200 μm. **(E)** Serum NEFA after 5 h food removal (*n* = 15–19). **(F)** Serum NEFA elevation after treatment of beta3 adrenergic agonist CL 316,243 stimulation for 20 min (*n* = 9–11). **(G)** Serum triglyceride levels in HFD-fed adipose-Piezo1^−/−^ and control mice (*n* = 6–10). All data were collected from male mice. ^*^P < 0.05, ^***^P < 0.001 vs. control mice. Data are expressed as mean ± SEM. SVF, stromal vascular fraction; pgWAT, perigonadal white adipose tissue; scWAT, subcutaneous white adipose tissue; BAT, brown adipose tissue; HFD, high-fat diet; NEFA, non-esterified fatty acids.

Despite no difference in body weight (Figure 2H), pgWAT mass was reduced in HFD-fed adipose-Piezo1^−/−^ mice compared with controls (Figures 3A,B). scWAT mass was not altered (Figure 3A). The pgWAT adipocyte size tended to be decreased (Figures 3C,D). The basal and beta3 adrenergic agonist-stimulated lipolysis were enhanced in adipose-Piezo1^−/−^ mice (Figures 3E,F). Furthermore, serum triglyceride levels also tended to be higher in adipose-Piezo1^−/−^ mice (Figure 3G). Therefore, Piezo1 appears to be necessary for adipocyte plasticity and fat storage in pgWAT of obese mice.

### Adipose-Specific Piezo1 Knockout Mice Develop Fatty Liver

**Figure 4:**
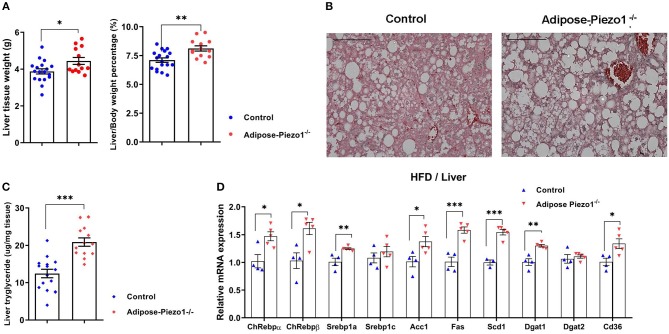
Effects of adipose Piezo1 knockout on hepatic lipid metabolism. **(A)** Liver weight and liver/body weight ratio of HFD-fed adipose-Piezo1^−/−^ and control mice. **(B)** Liver H&E staining (200X). Scale Bar, 200 μm. **(C)** Hepatic triglyceride contents. **(D)** mRNA expression of genes involved in lipogenesis [*n* = 13–18 for **(A,C)**. *n* = 4–5 for **(D)**]. All data were collected from male mice.^*^P < 0.05, ^**^P < 0.01, ^***^P < 0.001 vs. control mice, Data are expressed as mean ± SEM. HFD, high-fat diet.

Interestingly, adipose Piezo1 knockdown in mice fed a HFD increased liver weight (Figure 4A), likely due to the development of fatty liver (Figure 4B). Liver triglyceride contents were elevated in HFD-fed adipose-Piezo1^−/−^ mice (Figure 4C). Consistently, hepatic lipogenic genes including carbohydrate-response element-binding protein alpha (Chrebpα) and beta (Chrebpβ), sterol regulatory element binding protein-1a (Srebp-1a), acetyl-CoA carboxylase1 (Acc1), fatty acid synthase (Fas), stearoyl-CoA desaturase1 (Scd1), and diacylglycerol acyltransferase 1 (Dgat-1) were increased in adipose-Piezo1^−/−^ mice fed a HFD. In addition, the fatty acid transporter Cd36 was also elevated (Figure 4D). These data show that adipose Piezo1 knockdown causes lipid deposition in liver in obesity.

### Adipose Piezo1 Inhibition Induces Inflammation and Lipolysis Genes

**Figure 5:**
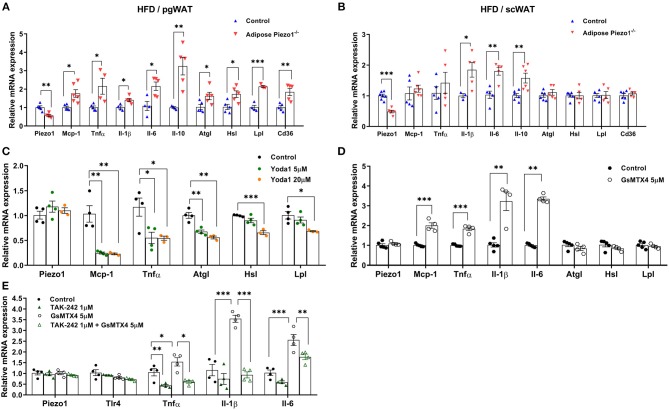
Piezo1 regulates adipose inflammation and lipolysis. **(A,B)** mRNA expression of Piezo1, IL-6, IL-1β, IL-10, TNFα, MCP-1, CD36, ATGL, HSL, LPL, and CD36 in pgWAT **(A)** and scWAT **(B)** of HFD-fed adipose-Piezo1^−/−^ and control mice, mRNA levels were normalized to Tbp, *n* = 4–7. **(C–E)** mRNA expression of proinflammatory and lipolytic genes in cultured primary adipocytes treated with the Piezo1 agonist Yoda1 **(C)**, Piezo1 antagonist GsMTx-4 **(D)**, or GsMTx-4 with the TLR4 inhibitor TAK-242 **(E)** (*n* = 3–5). All data were collected from male mice.^*^P < 0.05, ^**^P < 0.01, ^***^P < 0.001 vs. controls. *T*-test was used for **(A,B,D)** and one-way ANOVA was used for **(C,E)**. Data are from male mice and are expressed as mean ± SEM. pgWAT, perigonadal white adipose tissue; scWAT, subcutaneous white adipose tissue; HFD, high-fat diet.

The reduced pgWAT mass and fatty liver in adipose-Piezo1^−/−^ mice may represent mild lipodystrophy (30, 31). We measured the expression of key genes involved in the lipodystrophic syndrome. Plin1 and Cidec were mildly reduced while others were not altered in adipose tissue of adipose-Piezo1^−/−^ mice (Supplementary Figure 6A). The second possibility is that the larger adipocytes in adipose-Piezo1^−/−^ might undergo apoptosis. However, apoptotic markers were not changed in adipose tissue with Piezo1 knockdown (Supplementary Figure 6B). Furthermore, fatty acid transporter Cd36 was increased rather than decreased in pgWAT of adipose-Piezo1^−/−^ mice (Figure 5A). These results suggest that lipodystrophy, apoptosis and reduced fatty acid import are unlikely the cause of reduced pgWAT mass in the adipose-Piezo1^−/−^ mice.

Adipose plasticity has been linked to inflammation and lipolysis. Genes that regulate lipolysis including adipose tissue triglyceride lipase (Atgl), hormone-sensitive lipase (Hsl), and lipoprotein lipase (Lpl) were increased in the highly lipolytic pgWAT, but not in the less lipolytic scWAT of HFD-fed adipose-Piezo1^−/−^ mice (Figures 5A,B). Furthermore, pro-inflammatory cytokines including monocyte chemotactic protein-1 (Mcp-1), tumor necrosis factor-α (Tnfα), interleukin-1 β (Il-1β), and Il-6 were elevated in scWAT and pgWAT of the HFD-fed adipose-Piezo1^−/−^ mice (Figures 5A,B). Il-10, which suppresses the inflammatory response, was also induced (Figures 5A,B). pgWAT of the HFD-fed adipose-Piezo1^−/−^ mice appeared to contain more crown-like structures (Figure 3D). Although adipose inflammation is linked to fibrosis (32, 33), the expression of collagens and matrix metallopeptidases was not altered in adipose tissue with Piezo1 knockdown (Supplementary Figure 6C). The expression of the pro-inflammatory and lipolytic genes was not altered in the chow-fed adipose-Piezo1^−/−^ mice (not shown). The results suggest that Piezo1 is necessary for restraining pro-inflammatory response and lipolysis in obesity.

To investigate whether Piezo1 regulates inflammation directly in adipocytes, we activated or inhibited Piezo1 activity in cultured adipocytes. Treating adipocytes with the Piezo1 agonist Yoda1 reduced the expression of Mcp-1 and Tnfα. Lipolysis gene Atgl, Hsl, and Lpl were also mildly inhibited (Figure 5C). Conversely, treatment of adipocytes with the Piezo1 inhibitor GsMTx4 (34) elevated the expression of Mcp-1, Tnfα, Il-1β, and Il-6, but did not alter lipolytic genes (Figure 5D). TLR (Toll-like receptor) 4 signal pathway plays a major role in adipose pro-inflammatory cytokine expression. Treating adipocytes with TAK-242, a specific TLR4 inhibitor, abolished GsMTx4-induced Tnfα, Il-1β, and Il-6 expression (Figure 5E). These findings suggest that Piezo1 regulates TLR4-mediated cytokine expression in adipocytes.

## Discussion

Piezo1 is a transmembrane mechanically gated ion channel that is highly expressed in tissues with high mechanical pressure such as lungs, adipocytes, and blood vessels (14, 35). Adipocytes are under constant mechanical pressure from stored triglycerides inside and from extracellular matrix outside (36, 37). It is therefore not surprising that Piezo1 is highly expressed in adipocytes. Importantly, Piezo1 is upregulated in isolated adipocytes but not in the SVF of adipose tissue of obese mice, providing the initial evidence that Piezo1 may regulate adipocyte biological function.

The insulin resistance in the HFD-fed adipose-Piezo1^−/−^ mice indicates that the upregulation of adipose Piezo1 in obesity is likely adaptive. Piezo1 knockdown or inhibition enhances the expression of pro-inflammatory genes in adipocytes. This appears to be mediated by TLR4 since TLR4 inhibition abolishes pro-inflammatory cytokine expression induced by Piezo1 inhibition. TLR4 activation in adipocytes causes chronic inflammation and systemic insulin resistance (3, 38). However, how Piezo1 suppresses TLR4-mediated pro-inflammatory pathway in adipocytes remains elusive. Activation of Piezo1 allows nonselective membrane permeability of cations such as monovalent Na^+^, K^+^, and divalent Ca^2+^ and Mg^2+^ (39, 40). Piezo1-mediated calcium influx has been implicated in Piezo1 biological functions including regulation of red blood cell volume, apoptosis, myotube formation, and cell lineage choice(15, 18, 41–43). However, intracellular calcium is required for LPS-induced TLR4 activation at least in macrophages (44). Further studies are needed to investigate whether other cations such as Mg^2+^, which has been shown to inhibit TLR-stimulated TNF-a and IL6 production in monocytes (45), are involved in Piezo1-mediated inflammation suppression.

The smaller pgWAT mass and fatty liver in HFD-fed adipose-Piezo1^−/−^ mice suggest that Piezo1 plays an important role in maintaining adipose plasticity to prevent ectopic lipid deposition in liver. Our data show that the reduced adipocyte size in adipose-Piezo1^−/−^ mice is less likely caused by decreased fatty acid uptake, increased apoptosis or altered lipodystrophic genes. Rather adipose inflammation in adipose-Piezo1^−/−^ mice may enhance lipolysis and reduce adipocyte size (46, 47). This may also explain the increased hepatic lipogenesis and fatty liver in adipose-Piezo1^−/−^ mice (48, 49). Interestingly, the phenotype of smaller adipocytes and insulin resistance in adipose-Piezo1^−/−^ mice is similar to that in the volume-sensitive ion channel Swell1 knockout mice (50), although the underlying mechanisms may be different. Swell1 interacts with growth factor receptor-bound 2 (GRB2) and regulates insulin signaling. Our attempts to immunoprecipitate Piezo1-tdTomato to investigate its potential interaction with TLR4 or other proteins was unsuccessful (not shown), likely because Piezo1 is a 286 kDa membrane protein with 30–40 transmembrane domains (51, 52).

In summary, our studies add Piezo1 to a growing list of ion channels that are implicated in regulating insulin sensitivity, glucose metabolism and energy expenditure in obesity (53). We found that Piezo1 is highly expressed in adipocytes and the expression is regulated in obesity. Importantly, Piezo1 inhibition in adipocytes promotes TLR4-mediated inflammation, decreases adipocyte size, and induces insulin resistance. These results provide the first evidence that Piezo1, a novel mechanical sensitive ion channel, may play an important role in regulating adipose plasticity and insulin resistance.

## Data Availability

This manuscript contains previously unpublished data. The name of the repository and accession number are not available.

## Ethics Statement

This study was carried out in accordance with the recommendations of the Institutional Animal Care and Use Committee of the University of California, Irvine. The protocol was approved by the Institutional Animal Care and Use Committee of the University of California, Irvine.

## Author Contributions

CZ, QS, LT, YC, and JN conducted experiments and analyzed results. CZ and QY wrote the manuscript. MP, XL, and QY conceived the experimental design and supervised the studies.

### Conflict of Interest Statement

The authors declare that the research was conducted in the absence of any commercial or financial relationships that could be construed as a potential conflict of interest.

## References

[1] Reilly SM Saltiel AR. Adapting to obesity with adipose tissue inflammation. Nat Rev Endocrinol. (2017) 13:633–43. 10.1038/nrendo.2017.90 28799554

[2] Sun K Kusminski CM Scherer PE. Adipose tissue remodeling and obesity. J Clin Invest. (2011) 121:2094–101. 10.1172/JCI45887 21633177 PMC3104761

[3] Yang Q Vijayakumar A Kahn BB. Metabolites as regulators of insulin sensitivity and metabolism. Nat Rev Mol Cell Biol. (2018) 19:654–72. 10.1038/s41580-018-0044-8 30104701 PMC6380503

[4] Ray I Mahata SK De RK. Obesity: an immunometabolic perspective. Front Endocrinol. (2016) 7:157. 10.3389/fendo.2016.00157 28018292 PMC5149556

[5] Avgerinos KI Spyrou N Mantzoros CS Dalamaga M. Obesity and cancer risk: emerging biological mechanisms and perspectives. Metabolism. (2019) 92:121–35. 10.1016/j.metabol.2018.11.001 30445141

[6] Einarson TR Acs A Ludwig C Panton UH. Prevalence of cardiovascular disease in type 2 diabetes: a systematic literature review of scientific evidence from across the world in 2007-2017. Cardiovasc Diabetol. (2018) 17:83. 10.1186/s12933-018-0728-6 29884191 PMC5994068

[7] Perseghin G Calori G Lattuada G Ragogna F Dugnani E Garancini MP. Insulin resistance/hyperinsulinemia and cancer mortality: the Cremona study at the 15th year of follow-up. Acta Diabetol. (2012) 49:421–8. 10.1007/s00592-011-0361-2 22215126

[8] Laakso M. Cardiovascular disease in type 2 diabetes from population to man to mechanisms: the Kelly West Award Lecture 2008. Diabetes Care. (2010) 33:442–9. 10.2337/dc09-0749 20103560 PMC2809299

[9] Scherer PE. The many secret lives of adipocytes: implications for diabetes. Diabetologia. (2019) 62:223–32. 10.1007/s00125-018-4777-x 30465066 PMC6324990

[10] Vegiopoulos A Rohm M Herzig S. Adipose tissue: between the extremes. EMBO J (2017) 36:1999–2017. 10.15252/embj.201696206 28623240 PMC5509999

[11] Smorlesi A Frontini A Giordano A Cinti S. The adipose organ: white-brown adipocyte plasticity and metabolic inflammation. Obes Rev. (2012) 13(Suppl. 2):83–96. 10.1111/j.1467-789X.2012.01039.x 23107262

[12] Voglis G Tavernarakis N Mechanotransduction in the nematode *Caenorhabditis elegans*. In: Mechanosensitivity in Cells and Tissues, Kamkin A Kiseleva I, editors. Moscow: Academia Publishing House Ltd (2005). 21290765

[13] Bagriantsev SN Gracheva EO Gallagher PG. Piezo proteins: regulators of mechanosensation and other cellular processes. J Biol Chem. (2014) 289:31673–81. 10.1074/jbc.R114.612697 25305018 PMC4231648

[14] Hyman AJ Tumova S Beech DJ. Piezo1 channels in vascular development and the sensing of shear stress. Curr Top Membr. (2017) 79:37–57. 10.1016/bs.ctm.2016.11.001 28728823

[15] Kang H Hong Z Zhong M Klomp J Bayless KJ Mehta D. Piezo1 mediates angiogenesis through activation of MT1-MMP signaling. Am J Physiol Cell Physiol. (2019) 316:C92–103. 10.1152/ajpcell.00346.2018 30427721 PMC6383143

[16] Allison SJ. Hypertension: mechanosensation by PIEZO1 in blood pressure control. Nat Rev Nephrol. (2017) 13:3. 10.1038/nrneph.2016.165 27840417

[17] Albuisson J Murthy SE Bandell M Coste B Louis-Dit-Picard H Mathur J. Dehydrated hereditary stomatocytosis linked to gain-of-function mutations in mechanically activated PIEZO1 ion channels. Nat Commun. (2013) 4:1884. 10.1038/ncomms2899 23695678 PMC3674779

[18] Ma S Cahalan S LaMonte G Grubaugh ND Zeng W Murthy SE. Common PIEZO1 allele in African populations causes RBC dehydration and attenuates plasmodium infection. Cell. (2018) 173:443–55.e12. 10.1016/j.cell.2018.02.047 29576450 PMC5889333

[19] Etem EO Ceylan GG Ozaydin S Ceylan C Ozercan I Kuloglu T. The increased expression of Piezo1 and Piezo2 ion channels in human and mouse bladder carcinoma. Adv Clin Exp Med. (2018) 27:1025–31. 10.17219/acem/71080 30010255

[20] Chen X Wanggou S Bodalia A Zhu M Dong W Fan JJ. A feedforward mechanism mediated by mechanosensitive ion channel PIEZO1 and tissue mechanics promotes glioma aggression. Neuron. (2018) 100:799–815.e7. 10.1016/j.neuron.2018.09.046 30344046

[21] Li C Rezania S Kammerer S Sokolowski A Devaney T Gorischek A. Piezo1 forms mechanosensitive ion channels in the human MCF-7 breast cancer cell line. Sci Rep. (2015) 5:8364. 10.1038/srep08364 25666479 PMC4322926

[22] Folch J Lees M Sloane Stanley GH. A simple method for the isolation and purification of total lipides from animal tissues. J Biol Chem. (1957) 226:497–509. 13428781

[23] Parlee SD Lentz SI Mori H MacDougald OA. Quantifying size and number of adipocytes in adipose tissue. Methods Enzymol. (2014) 537:93–122. 10.1016/B978-0-12-411619-1.00006-9 24480343 PMC4069255

[24] Ranade SS Qiu Z Woo SH Hur SS Murthy SE Cahalan SM. Piezo1, a mechanically activated ion channel, is required for vascular development in mice. Proc Natl Acad Sci USA. (2014) 111:10347–52. 10.1073/pnas.1409233111 24958852 PMC4104881

[25] Coste B Murthy SE Mathur J Schmidt M Mechioukhi Y Delmas P. Piezo1 ion channel pore properties are dictated by C-terminal region. Nat Commun. (2015) 6:7223. 10.1038/ncomms8223 26008989 PMC4445471

[26] Ilkan Z Wright JR Goodall AH Gibbins JM Jones CI Mahaut-Smith MP Evidence for shear-mediated Ca(2+) entry through mechanosensitive cation channels in human platelets and a megakaryocytic cell line. J Biol Chem. (2017) 292:9204–17. 10.1074/jbc.M116.766196 28416610 PMC5454102

[27] Wollscheid B Bausch-Fluck D Henderson C O'Brien R Bibel M Schiess R. Mass-spectrometric identification and relative quantification of N-linked cell surface glycoproteins. Nat Biotechnol. (2009) 27:378–86. 10.1038/nbt.1532 19349973 PMC2829300

[28] Yuan F Zhang L Cao Y Gao W Zhao C Fang Y. Spermidine/spermine N1-acetyltransferase-mediated polyamine catabolism regulates beige adipocyte biogenesis. Metabolism. (2018) 85:298–304. 10.1016/j.metabol.2018.04.007 29715464 PMC7269456

[29] Cahalan SM Lukacs V Ranade SS Chien S Bandell M Patapoutian A. Piezo1 links mechanical forces to red blood cell volume. Elife. (2015) 4:e07370. 10.7554/eLife.07370 26001274 PMC4456639

[30] Huang-Doran I Sleigh A Rochford JJ O'Rahilly S Savage DB. Lipodystrophy: metabolic insights from a rare disorder. J Endocrinol. (2010) 207:245–55. 10.1677/JOE-10-0272 20870709

[31] Kumar S Samaras K. The impact of weight gain during HIV treatment on risk of pre-diabetes, diabetes mellitus, cardiovascular disease, and mortality. Front Endocrinol. (2018) 9:705. 10.3389/fendo.2018.00705 30542325 PMC6277792

[32] Luo T Nocon A Fry J Sherban A Rui X Jiang B. AMPK activation by metformin suppresses abnormal extracellular matrix remodeling in adipose tissue and ameliorates insulin resistance in obesity. Diabetes. (2016) 65:2295–310. 10.2337/db15-1122 27207538 PMC4955985

[33] Sun K Tordjman J Clement K Scherer PE. Fibrosis and adipose tissue dysfunction. Cell Metab. (2013) 18:470–7. 10.1016/j.cmet.2013.06.016 23954640 PMC3795900

[34] Romac JM Shahid RA Swain SM Vigna SR Liddle RA. Piezo1 is a mechanically activated ion channel and mediates pressure induced pancreatitis. Nat Commun. (2018) 9:1715. 10.1038/s41467-018-04194-9 29712913 PMC5928090

[35] Zhong M Komarova Y Rehman J Malik AB. Mechanosensing Piezo channels in tissue homeostasis including their role in lungs. Pulm Circ. (2018) 8:2045894018767393. 10.1177/2045894018767393 29521167 PMC6024292

[36] Lapid K Graff JM. Form(ul)ation of adipocytes by lipids. Adipocyte. (2017) 6:176–86. 10.1080/21623945.2017.1299298 28425847 PMC5638366

[37] Shoham N Gefen A. Mechanotransduction in adipocytes. J Biomech. (2012) 45:1–8. 10.1016/j.jbiomech.2011.10.023 22112919

[38] Shi H Kokoeva MV Inouye K Tzameli I Yin H Flier JS. TLR4 links innate immunity and fatty acid-induced insulin resistance. J Clin Invest. (2006) 116:3015–25. 10.1172/JCI28898 17053832 PMC1616196

[39] Wu J Lewis AH Grandl J. Touch, tension, and transduction - the function and regulation of piezo ion channels. Trends Biochem Sci. (2017) 42:57–71. 10.1016/j.tibs.2016.09.004 27743844 PMC5407468

[40] Gnanasambandam R Gottlieb PA Sachs F. The Kinetics and the permeation properties of piezo channels. Curr Top Membr. (2017) 79:275–307. 10.1016/bs.ctm.2016.11.004 28728821

[41] Gudipaty SA Lindblom J Loftus PD Redd MJ Edes K Davey CF. Mechanical stretch triggers rapid epithelial cell division through Piezo1. Nature. (2017) 543:118–21. 10.1038/nature21407 28199303 PMC5334365

[42] Pathak MM Nourse JL Tran T Hwe J Arulmoli J Le DT. Stretch-activated ion channel Piezo1 directs lineage choice in human neural stem cells. Proc Natl Acad Sci USA. (2014) 111:16148–53. 10.1073/pnas.1409802111 25349416 PMC4234578

[43] Tsuchiya M Hara Y Okuda M Itoh K Nishioka R Shiomi A. Cell surface flip-flop of phosphatidylserine is critical for PIEZO1-mediated myotube formation. Nat Commun. (2018) 9:2049. 10.1038/s41467-018-04436-w 29799007 PMC5967302

[44] Chiang CY Veckman V Limmer K David M. Phospholipase Cgamma-2 and intracellular calcium are required for lipopolysaccharide-induced Toll-like receptor 4 (TLR4) endocytosis and interferon regulatory factor 3 (IRF3) activation. J Biol Chem. (2012) 287:3704–9. 10.1074/jbc.C111.328559 22158869 PMC3281733

[45] Sugimoto J Romani AM Valentin-Torres AM Luciano AA Ramirez Kitchen CM Funderburg N. Magnesium decreases inflammatory cytokine production: a novel innate immunomodulatory mechanism. J Immunol. (2012) 188:6338–46. 10.4049/jimmunol.1101765 22611240 PMC3884513

[46] Cani PD Amar J Iglesias MA Poggi M Knauf C Bastelica D. Metabolic endotoxemia initiates obesity and insulin resistance. Diabetes. (2007) 56:1761–72. 10.2337/db06-1491 17456850

[47] Zu L He J Jiang H Xu C Pu S Xu G. Bacterial endotoxin stimulates adipose lipolysis via toll-like receptor 4 and extracellular signal-regulated kinase pathway. J Biol Chem. (2009) 284:5915–26. 10.1074/jbc.M807852200 19122198

[48] Shulman GI Ectopic fat in insulin resistance, dyslipidemia, and cardiometabolic disease. N Engl J Med. (2014) 371:1131–41. 10.1056/NEJMra1011035 25229917

[49] Liu L Mei M Yang S Li Q. Roles of chronic low-grade inflammation in the development of ectopic fat deposition. Mediators Inflamm. (2014) 2014:418185. 10.1155/2014/418185 25143667 PMC4131072

[50] Zhang Y Xie L Gunasekar SK Tong D Mishra A Gibson WJ SWELL1 is a regulator of adipocyte size, insulin signalling and glucose homeostasis. Nat Cell Biol. (2017) 19:504–17. 10.1038/ncb3514 28436964 PMC5415409

[51] Coste B Mathur J Schmidt M Earley TJ Ranade S Petrus MJ. Piezo1 and Piezo2 are essential components of distinct mechanically activated cation channels. Science. (2010) 330:55–60. 10.1126/science.1193270 20813920 PMC3062430

[52] Saotome K Murthy SE Kefauver JM Whitwam T Patapoutian A Ward AB. Structure of the mechanically activated ion channel Piezo1. Nature. (2018) 554:481–6. 10.1038/nature25453 29261642 PMC6010196

[53] Vasconcelos LH Souza IL Pinheiro LS Silva BA. Ion channels in obesity: pathophysiology and potential therapeutic targets. Front Pharmacol. (2016) 7:58. 10.3389/fphar.2016.00058 27065858 PMC4811910

