# Supplementary material for: Mechanosensitive Ion Channel Piezo1 Regulates Diet-Induced Adipose Inflammation and Systemic Insulin Resistance
Source: Front Endocrinol (Lausanne). 2019 Jun 13;10:373. doi: 10.3389/fendo.2019.00373 (PMC6584899; doi:10.3389/fendo.2019.00373)
Supplement: Supplementary file 1 [file Data_Sheet_1.PDF]

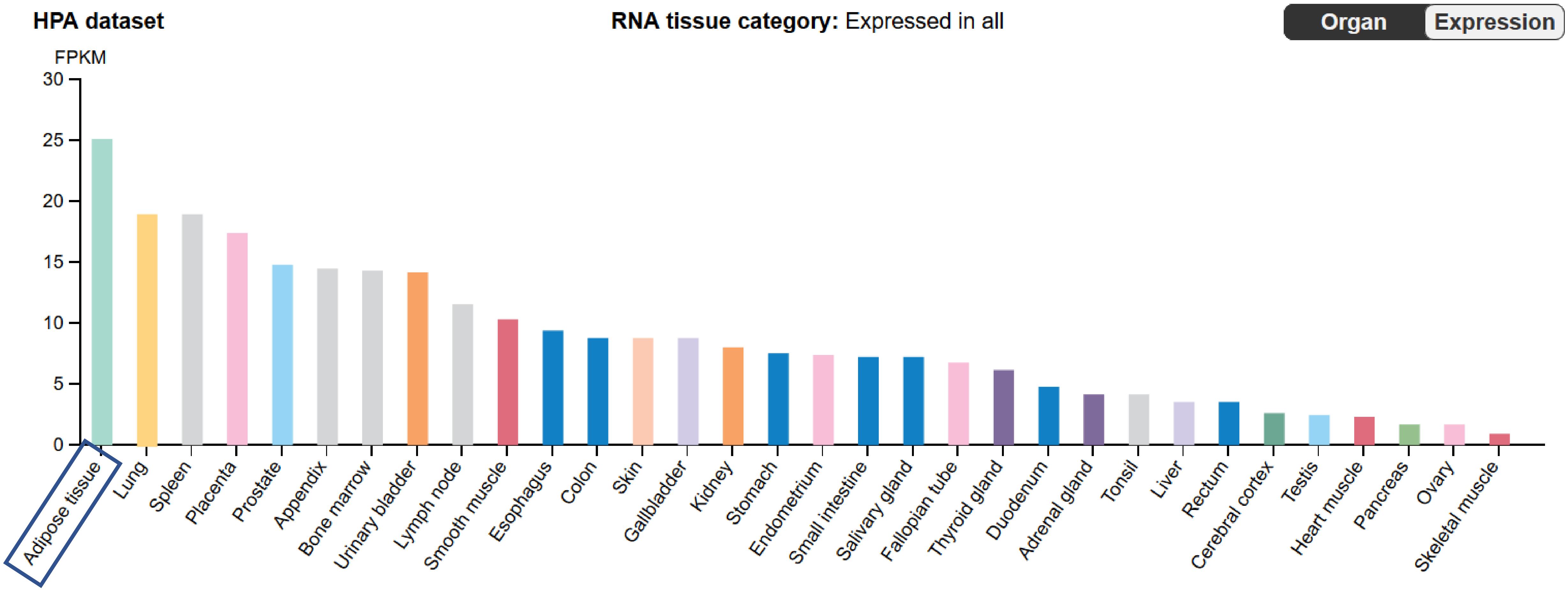

Supplementary Figure 1. Piezo1 mRNA levels in human tissues from Atlas RNA-seq data.

Profile GDS3142 / 1455157_a_at
Title Various tissues
Organism Mus musculus

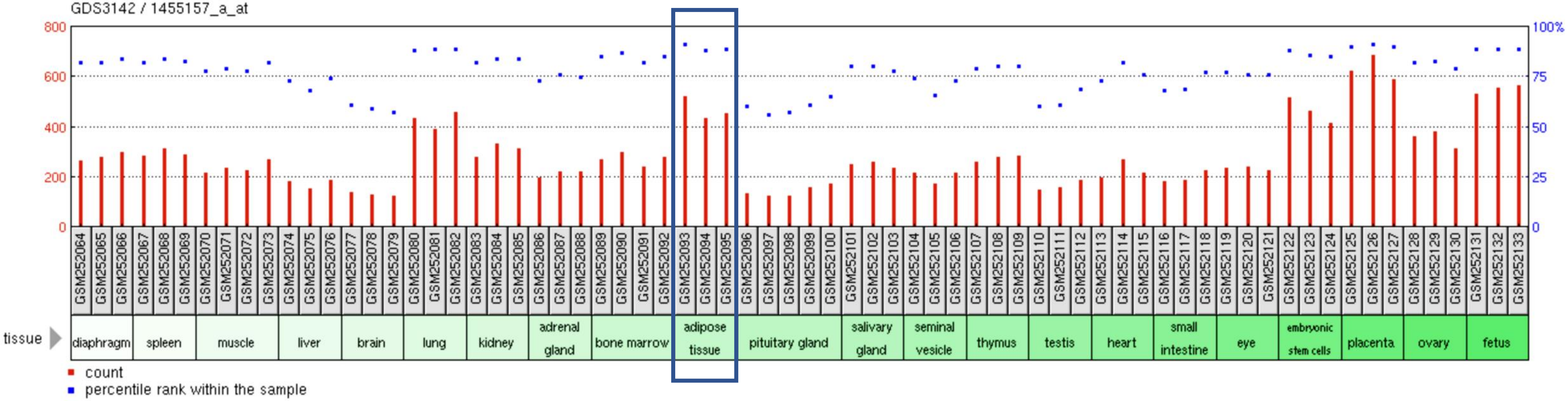

Supplementary Figure 2. Piezo1 mRNA levels in mouse tissues (GEO Profiles ID: 49893452).

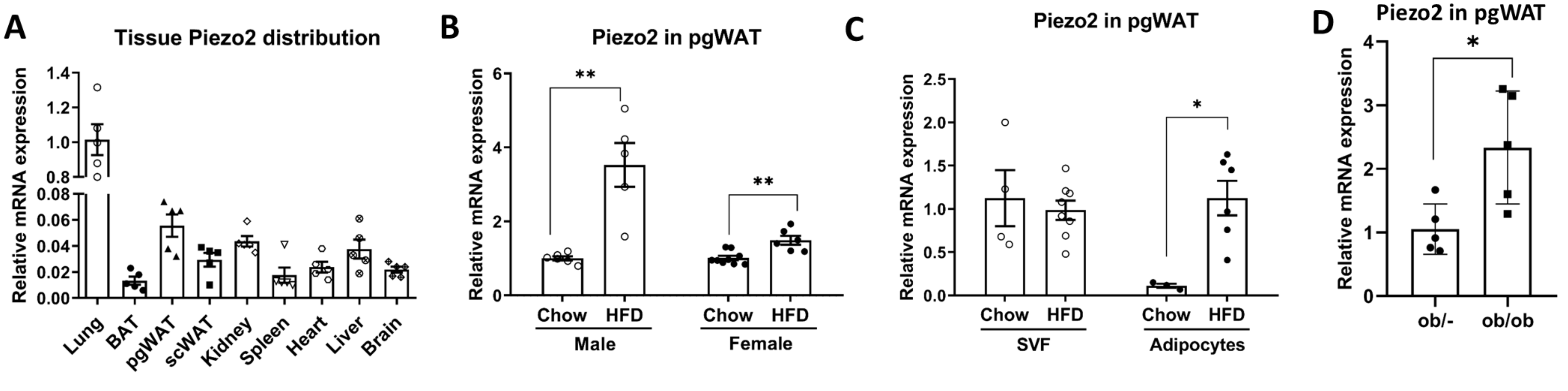

Supplementary Figure 3. Piezo2 tissue distribution and adipose expression. (A) Piezo2 mRNA expression in male C57BL/6 mice fed a chow diet.（n=5） **(B)** Piezo2 mRNA expression in pgWAT of chow-fed and high-fat-diet-fed mice. (n=5-9) **(C)** Piezo2 mRNA in adipocytes and SVF isolated from pgWAT of mice fed a chow or high-fat diet. (n=3-8) **(D)** Piezo2 mRNA expression in pgWAT of ob/ob and control ob/- mice. (n=5-6). The expression levels were corrected with Tbp. *,P<0.05 **,P<0.01 versus chow-diet. Data are expressed as mean ± SEM. SVF: stromal vascular fraction; pgWAT: perigonadal white adipose tissue; scWAT: subcutaneous white adipose tissue; BAT: brown adipose tissue; HFD: high fat diet.

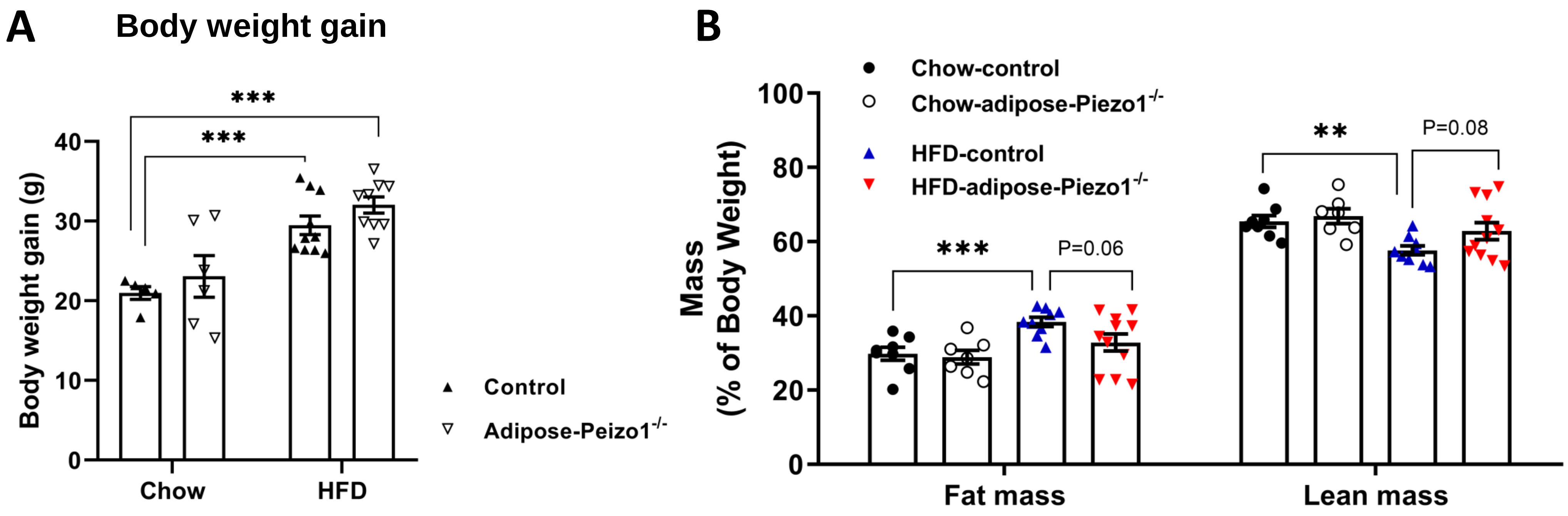

Supplementary Figure 4. Body weight gain and composition. (A) Body weight gain when the mice were sacrificed. **(B)** Lean and fat mass were measured by an EchoMRI system and corrected to the body weight. n=6-11 per group. *,$P<0.05$ **,$P<0.01$ ***,$P<0.001$ versus chow-diet controls. Data are expressed as mean ± SEM.

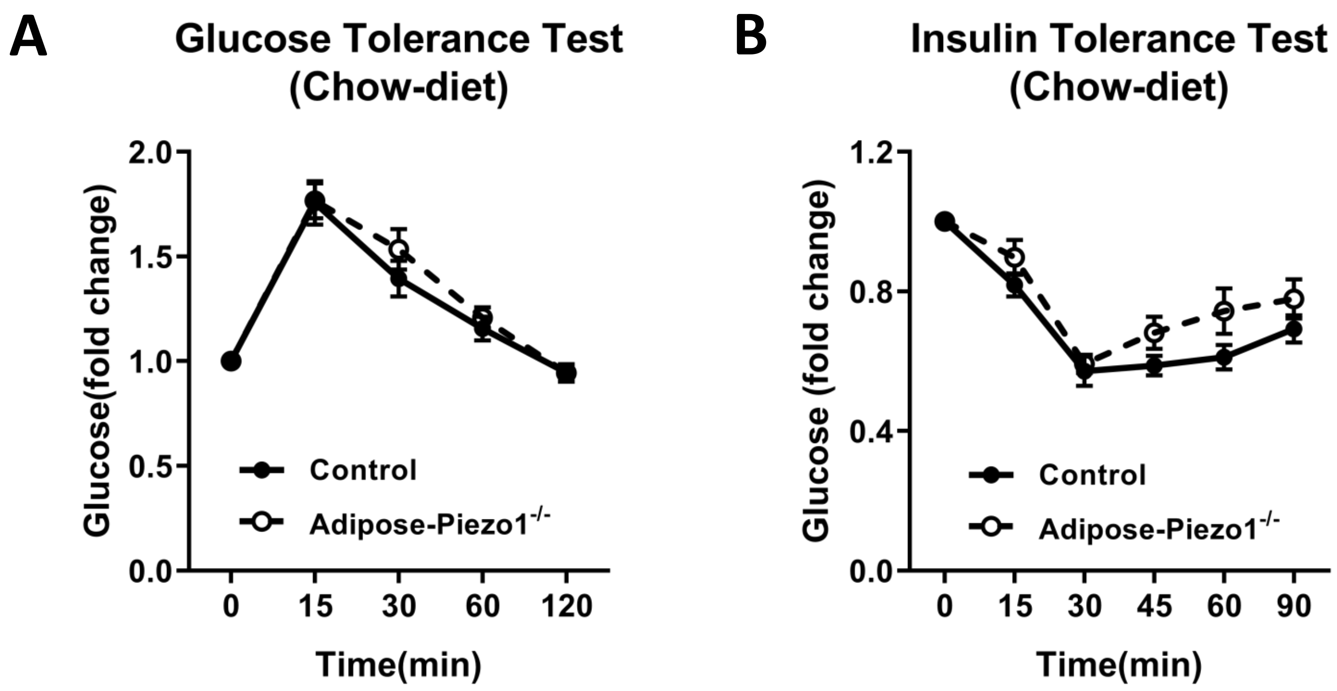

Supplementary Figure 5. Glucose fold changes in the glucose and insulin tolerance tests. Glucose was corrected to basal levels in the glucose tolerance test **(A)** and insulin tolerance test **(B)** using data from Figure 2D and 2E. n=8-10 per condition, data are expressed as mean ± SEM.

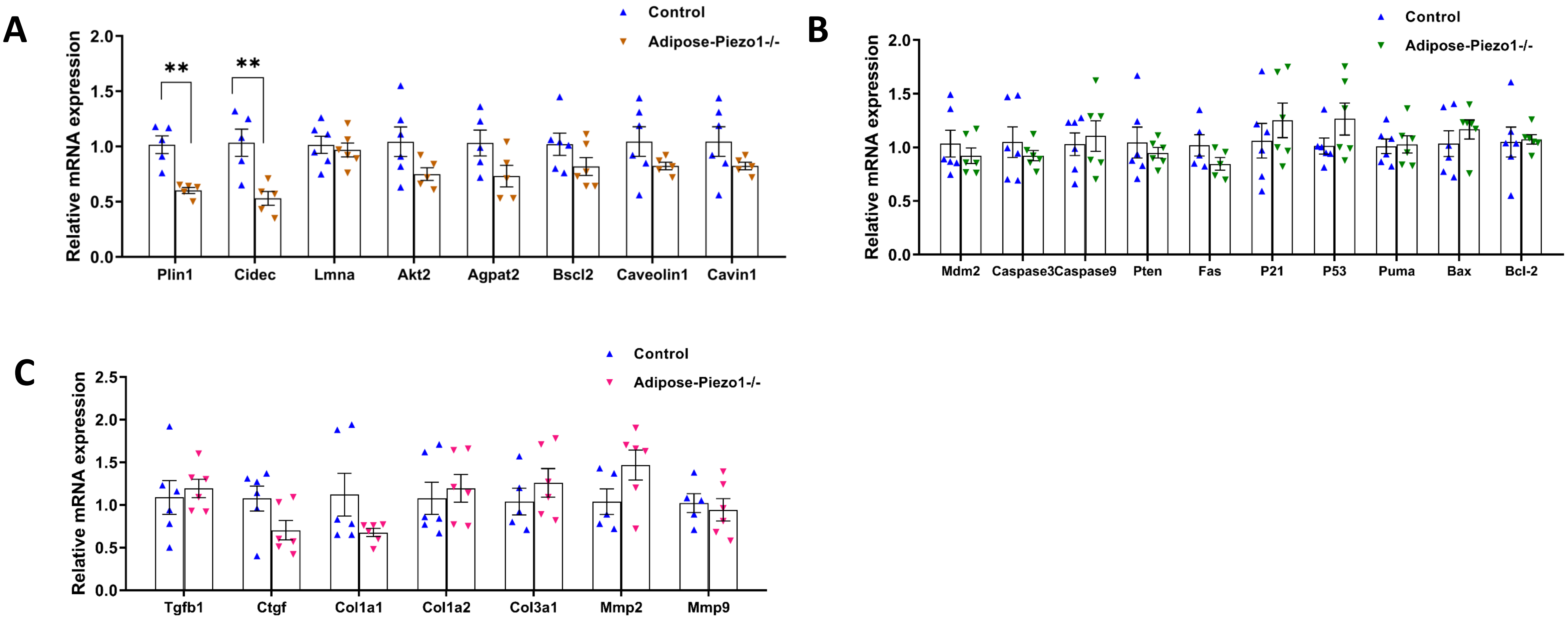

Supplementary Figure 6. Effects of Piezo1 knockdown on lipodystrophy, apoptosis and fibrosis. Expression of **(A)** Lipodytrophy-related genes (normalized with Tbp); **(B)** Apoptosis-related genes (normalized with Tbp); **(C)** Fibrosis related genes (normalized with Tbp) in pgWAT of HFD-fed adipose-Piezo1-/- and control mice. n=5-6, *P<0.05 versus control mice. Data are expressed as mean ± SEM. pgWAT: perigonadal white adipose tissue; HFD: high fat diet.
