# Supplementary material for: Mechanosensitive Ion Channel Piezo1 Regulates Diet-Induced Adipose Inflammation and Systemic Insulin Resistance
Source: Front Endocrinol (Lausanne). 2019 Jun 13;10:373. doi: 10.3389/fendo.2019.00373 (PMC6584899; doi:10.3389/fendo.2019.00373)
Supplement: Supplementary file 2 [file Data_Sheet_2.PDF]

Supplementary Table 1: Primers for qPCR.

Gene	Forward Primer	Reverse Primer
Piezo1	TACCAGTACCTGCTGTGTTTG	GTAGGTACAGCCACTTGATGAG
Piezo2	AGAGTCGGAAAGAGATACCCTC	CCAGACGATACAGATGAGAAGGA
ChRebpα	CGACACTCACCCACCTCTTC	TTGTTCAGCCGGATCTTGTC
ChRebpβ	TCTGCAGATCGCGTGGAG	CTTGTCCCGGCATAGCAAC
Srebp1a	TTGGCACCTGGGCTGCT	GCGCCATGGACGAGCTG
Srebp1c	GAGCCATGGATTGCACATTT	CTCAGGAGAGTTGGCACCTG
Acc1	TGTACAAGCAGTGTGGGCTGGCT	CCACATGGCCTGGCTTGGAGGG
Fas	GCTGCGGAAACTTCAGGAAAT	AGAGACGTGTCACTCCTGGACTT
Ssd1	TTCTTGCGATACACTCTGGTGC	CGGGATTGAATGTTCTTGTCGT
Dgat1	TCCGTCCAGGGTGGTAGTG	TGAACAAAGAATCTTGCAGACGA
Dgat2	GCGCTACTTCCGAGACTACTT	GGGCCTTATGCCAGGAAACT
Cd36	AACTTGTGGCCTTGCACTCT	GCAGAATCAAGGGAGAGCA
Mcp-1	GCATCTGCCCTAAGGTCTTCA	TGCTTGAGGTGGTTGTGGAA
Tnfα	ACCCTGGTATGAGCCCATATAC	ACACCCATTCCCTTCACAGAG
Il-1β	ATGAGAGCATCCAGCTTCAA	TGAAGGAAAAGAAGGTGCTC
Il-6	GAGGATACCACTCCCAACAGA	AAGTGCATCATCGTTGTTCAT
Il-10	GCTCTTACTGACTGGCATGAG	CGCAGCTCTAGGAGCATGTG
Atgl	CAACGCCACTCACATCTACGG	GGACACCTCAATAATGTTGGCAC
Hsl	CCAGCCTGAGGGCTTACTG	CTCCATTGACTGTGACATCTCG
Lpl	GCCCAGCAACATTATCCAGT	GGTCAGACTTCCTGCTACGC
Tlr4	ATGGCATGGCTTACACCACC	GAGGCCAATTTTGTCTCCACA
Plin1	GGGACCTGTGAGTGCTTCC	GTATTGAAGAGCCGGGATCTTTT
Cidec	ATGGACTACGCCATGAAGTCT	CGGTGCTAACACGACAGGG
Lmna	ACCCCGCTGAGTACAACCT	TTCGAGTGACTGTGACACTGG
Akt2	ACGTGGTGAATACATCAAGACC	GCTACAGAGAAATTGTTCAGGGG
Apgat2	CAGCCAGGTTCTACGCCAAG	TGATGCTCATGTTATCCACGGT
Bscl2	TGGGGCAAGAGAGACATGC	TCTTCCACAGGGACGATACCC
Caveolin1	ATGTCTGGGGGCAAATACGTG	CGCGTCATACACTTGCTTCT
Cavin1	CAGCGTCAACGTGAAGACC	CCTCCGACTCTTTCAGCGAC
Mdm2	TGTCTGTGTCTACCGAGGGTG	TCCAACGGACTTTAACAACTTCA
Caspase3	ATGGAGAACAACAAAACCTCAGT	TTGCTCCCATGTATGGTCTTTAC
Caspase9	TCCTGGTACATCGAGACCTTG	AAGTCCCTTTCGCAGAAACAG
Pten	TGGATTCGACTTAGACTTGACCT	GCGGTGTCATAATGTCTCTCAG
P21	CCTGGTGATGTCCGACCTG	CCATGAGCGCATCGCAATC
P53	CTCTCCCCCGCAAAAGAAAAA	CGGAACATCTCGAAGCGTTTA
Puma	AGCAGCACTTAGAGTCGCC	CCTGGGTAAGGGGAGGAGT
Bax	TGAAGACAGGGGCCTTTTTG	AATTCGCCGGAGACACTCG
Bcl-2	ATGCCTTTGTGGAACTATATGGC	GGTATGCACCCAGAGTGATGC
Tgfb1	CTCCCGTGGCTTCTAGTGC	GCCTTAGTTTGGACAGGATCTG

Ctgf	GGGCCTCTTCTGCGATTTC	ATCCAGGCAAGTGCATTGGTA
Col1a1	GCTCCTCTTAGGGGCCACT	CCACGTCTCACCATTGGGG
Col1a2	GTAACTTCGTGCCTAGCAACA	CCTTTGTCAGAATACTGAGCAGC
Mmp2	CAAGTTCCCCGCCGATGTC	TTCTGGTCAAGGTCACCTGTC
Mmp9	CTGGACAGCCAGACACTAAAG	CTCGCGGCAAGTCTTCAGAG
Tbp	CCCTATCACTCCTGCCACAC	ACGAAGTGCAATGGTCTTTAG
36b4	CCCTGAAGTGCTCGACATCA	TGCGGACACCCTCCAGAA
